# Triploid Atlantic salmon (*Salmo salar*) may have increased risk of primary field outbreaks of infectious salmon anaemia

**DOI:** 10.1111/jfd.13695

**Published:** 2022-08-01

**Authors:** Arnfinn Aunsmo, Lisbeth Martinsen, Torkjel Bruheim, Mats Martin Sekkelsten‐Kindt, Ane Sandtrø, Solveig Gaasø, Stine Braaen, Espen Rimstad

**Affiliations:** ^1^ Department of Production Animal Clinical Sciences, Faculty of Veterinary Medicine Norwegian University of Life Sciences Ås Norway; ^2^ Laxar Fiskeldi Reykjavik Iceland; ^3^ Norway Royal Salmon Trondheim Norway; ^4^ AquaGen AS Trondheim Norway; ^5^ PHARMAQ, Skogmo Industriområde Overhalla Norway; ^6^ Frøygruppen Sistranda Norway; ^7^ Department of Paraclinical Sciences Norwegian University of Life Sciences Ås Norway

**Keywords:** ISA, susceptibility, triploid, vaccination

## Abstract

The impact that escaped farmed fish may have on wild populations is of major concern for Atlantic salmon (*Salmo salar*) farming. Triploid fish, being infertile, were originally introduced to mitigate the genetic impact of escaped fish. In the recent years, an increase in the number of infectious salmon anaemia (ISA) outbreaks in Norway has been observed, mainly in the northern parts, which is also where farming of triploid fish has been licensed. The present study investigated the susceptibility of triploid Atlantic salmon to ISA both by field observations and experimental infections. Based on field observations, we found an increased susceptibility, with 9.4 increased odds to primary ISA outbreaks in triploid fish versus diploid fish at production‐site level, and a tendency of increased odds (3.4) of ISA in triploid fish at individual cage level at sited with primary outbreaks. At some sites, ISA outbreaks were only diagnosed in cages with triploid fish and not in cages with diploid fish. Primary ISA outbreaks are the source for further spread of the disease, and it is noteworthy that in an experimental trial we found significantly more viral RNA in non‐ISA‐vaccinated triploid than in non‐ISA‐vaccinated diploid fish at the peak of the infection. Interestingly, the notable differences of susceptibility to ISA for non‐ISA vaccinated diploid and triploid fish observed in field were not repeated experimentally. The possible increased risk of ISA should be considered when evaluating the costs and benefits of triploid salmon in farming. It is recommended to keep triploid and diploid fish in biosecure separated sites, or that triploid fish are not farmed at all.

## 1 INTRODUCTION

Farmed Atlantic salmon (*Salmo salar*) have been genetically selected for important commercial traits since the start of modern salmon farming. This may have reduced the adaption of farmed fish to the natural environment, and a major concern regarding the escape of farmed salmon is the potential gene flow from farmed salmon to wild salmon due to interbreeding (O'Sullivan et al., 2020). Production of infertile farmed fish would be an effective measure to eliminate such gene flow and could also mitigate pre‐harvest sexual maturation. Triploid salmon are infertile and licences to farm triploid fish have been issued by Norwegian authorities to stimulate their use in Atlantic salmon farming. Triploidization is obtained by high‐pressure treatment of newly fertilized salmon eggs. Triploid fish will, due to the extra set of chromosomes, have significantly larger cells than diploid fish. For many species, polyploidy increases body size, but fish compensate by reducing the number of cells and the individual body size appears normal and similar to that of diploid fish. However, triploid salmon differ from natural diploids by requiring more dietary phosphorus during early development, they may have a different gill microbiota, and they have a higher prevalence of eye cataracts (Brown et al., 2021; Olsvik et al., 2020; Sambraus et al., 2020). Studies of the relative susceptibility to infectious diseases for triploid versus diploid salmon have been limited; however, no differences were found for the susceptibility to infectious pancreatic necrosis virus (IPNV), salmonid alphavirus (SAV) or to severe *Neoparamoeba perurans* infestation (Chalmers et al., 2017; Moore et al., 2017). Also, vaccination of triploid salmon against furunculosis was found to give good protection (Chalmers et al., 2016). Winter ulcers have been reported to be prevalent in triploid fish that were moved to sea water during the fall (Stien et al., 2019).

The number of annual outbreaks of infectious salmon anaemia (ISA) in Norway has been low to moderate since a large ISA epidemic in the early 1990s. ISA can reduce the economical sustainability for an affected company, the virulent virus may further infect neighbouring farms, it is a notifiable fish disease and outbreaks may affect trade in salmon on the international market. A relatively sharp increase in the annual number of ISA outbreaks, i.e., 23 outbreaks, was recorded in Norway in 2020 and 25 outbreaks were recorded in 2021. The increase in outbreaks has mainly occurred in the northern parts of Norway (Dean et al., 2022), which is the area where farming of triploid fish has been licensed. In an earlier study, an increased risk of primary ISA outbreaks was found when the production site was located at high latitude, i.e., in northern parts (Lyngstad et al., 2018). The proportion of salmon produced in the two northernmost counties has increased from 14% to about 22% of the total production of Norway over the last 20 years. Studies of infection, disease, and epidemiology of ISA in triploid fish are therefore needed.

The two phenotypic variants of ISA virus (ISAV), the ISAV‐HPR0 and ISAV‐HPRΔ, non‐virulent and virulent, respectively, are named after the highly polymorphic region (HPR) in the hemagglutinin‐esterase (HE) gene. The non‐virulent and virulent phenotypic ISAV variants are however, differentiated not only by sequence differences in the HPR of the HE gene, but also by differences in the fusion (F) gene.

The non‐virulent HPR0 strain has a strong tropism for gills and is highly prevalent in the sea phase of farmed salmon (Lyngstad et al., 2012). The origin of primary outbreaks of ISA is thought to be a transition from a non‐virulent HPR0 to a virulent HPRΔ variant. Outbreaks assumed to be associated with the transition from non‐virulent HPR0 to ISAV‐HPRΔ are often termed primary outbreaks (Christiansen et al., 2017; Nylund et al., 2019). Around 40% of ISA outbreaks in Norway have been considered to be primary outbreaks (Aldrin et al., 2021). If virulent ISAV‐HPRΔ from primary outbreaks spreads horizontally through live‐animal movements, equipment, or through passive transport via water currents, to nearby farms and causes disease outbreaks, then these outbreaks are termed secondary outbreaks (Lyngstad et al., 2018). In ISA epidemics, secondary outbreaks obviously predominate. Under field conditions, it is often not possible to trace the disease to a known source of infection, and the lack of definite tracking may be the basis for the classification as primary outbreak. Thus, the term primary outbreak in the field also often encompasses outbreaks in which the infection source cannot be identified. In recent years, many sporadic outbreaks of ISA in Norway have been classified as primary outbreaks (Dean et al., 2022).

In the present work, the epidemiology of ISA outbreaks in triploid fish in farms was studied. The results indicate that primary ISA outbreaks have a significantly higher probability of occurring in triploid fish than in diploids. To test the results found in the field study, an experimental susceptibility and vaccination trial was subsequently performed.

## 2 MATERIALS AND METHODS

### 2.1 Field outbreaks of ISA

A commercial farming company where production of triploid fish is a large part of total production, had 12 confirmed and two suspected (detection of ISAV‐HPRΔ by RT‐PCR but negative in all subsequent confirmative tests) ISA outbreaks in the period 2015–2020. These outbreaks were investigated in detail and made the basis for analysis of the specific risk factors for outbreaks of ISA in triploid fish. All production took place in northern Norway in production areas 10–12. An overview of the outbreaks can be found in Table S1 in Supplementary material.

#### 2.1.1 Fish stock

During the period 2015–2019, the company had 57 sea transfers into a total of 428 net cages. Diploid fish made up 71.3% and triploid 28.7% of stocked cages. The diploid and triploid fish were kept in the same farms but in separate cages. The fish originated from four different genetic breeds with diploid fish coming from all four breeds and triploid coming from two of those breeds. The fish were not vaccinated against ISA.

#### 2.1.2 Diagnostics and management of ISA cases

The fish sites of the company had monthly routine control by fish health professionals. Increased mortality led automatically to increased investigations of all cages. Suspicion of ISA due to clinical findings was investigated further by RT‐PCR analyses. Heart samples were submitted to PatoGen, Ålesund, Norway, a laboratory accredited for PCR analysis. ISA is a notifiable disease and suspicion of disease was reported to the Norwegian Food Safety Authority, which investigated a suspicion of ISA by their own sampling and analysis with RT‐PCR‐ and histopathologic examination. The regulatory criteria of the handling of the outbreaks were set by the Norwegian Food Safety Authority. In addition, the farming company monitored verified outbreaks by sampling for RT‐PCR of individual cages to manage the harvest order of the cages in a best possible way.

#### 2.1.3 Tracing and origin of ISA‐HPRΔ

Sequencing and phylogenetic analysis of ISAV‐HPRΔ had been performed by PatoGen, the diagnostic company that served the sites, as a part of their portfolio of analyses. Alignment of the nucleotide sequence of the gene segment encoding the HE gene was the basis of the phylogenetic analysis.

### 2.2 Vaccine and infection trials

#### 2.2.1 Experimental fish

**FIGURE 1:**
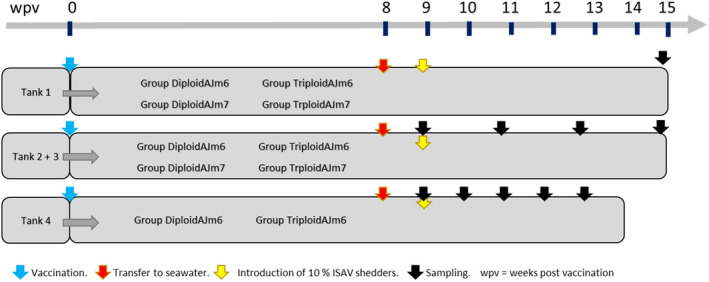
Design of vaccine and infection trials

The study included one experiment with initial vaccination and subsequent challenge, and one challenge experiment. Both were conducted at VESO Vikan aquatic research facility (Vikan, Norway, internal reference V4596). Diploid and triploid fish used in the study were both of SalmoBreed origin, confirmed free of known salmon pathogens and there was no difference of clinical presentation between triploid and diploid groups before initiation. The design of the study is shown in Figure 1.

Fish were fed according to standard procedures and kept at 12 C temperature during the experiment. The fish *n* = 527, size 40–60 g, were in fresh water at vaccination and were put on sea water one week before infection, i.e., at week 8 post vaccination (wpv). The experiment was performed with photoperiod 12:12, except for 2–8 wpv when the fish were exposed to 24:00 photoperiod to induce smoltification. Prior to handling, the fish were anaesthetised by bath immersion in benzocaine chloride (1–2 min, 60 mg/L). A concentration of benzocaine chloride of 120 mg/L was used to kill.

The fish were observed minimum once per day. The criteria used to assess humane endpoint were loss of ability to maintain their position in the water, darkening of skin, bleedings and exophthalmia and no evasion to netting. Fish that reached the terminal stage were killed. The water in all experimental tanks were changed from fresh water to sea water one week before ISAV‐shedder fish were added. The vaccination and experimental challenge were performed in compliance with the regulatory requirements by Norwegian Food Safety Authority, EU Council Directive 2004/10/EC and Guidelines to Good Manufacturing Practice by European Commission Directives 2003/94/EC and 91/412/EC. The Norwegian Food Safety Authority (NFDA) according to the European Union Directive 2010/63/EU for animal experiments approved the experiment.

A pre‐infection test with three different doses of ISAV, strain AL V321 injected i.p., was performed with diploid fish, 10 fish per group, to estimate susceptibility (Kibenge et al., 2009). Mortality of injected fish began on day 12 at the highest dose, and based on the mortality curves, it was chosen to use the highest dose in the experiment itself.

#### 2.2.2 Vaccines

The fish were immunized with either a vaccine with specific ISA component (ALPHA JECT micro 7 ILA (AJm7), Pharmaq part of Zoetis, Norway) or a vaccine without an ISA component (ALPHA JECT micro 6 (AJm6), Pharmaq). The only difference between AJm6 and AJm7 is the ISAV component. Here, ISA‐vaccinated means vaccinated with AJm7, while non‐ISA‐vaccinated means vaccinated with AJm6. The different groups were marked by combinations of clipping of right or left maxilla and adipose fin.

***Tank 1*** was a vaccine trial where mortality of vaccinated diploid and triploid salmon was measured after exposure to the ISAV and consisted of 30 fish in each of the groups Triploid AJm6, Triploid AJm7, Diploid AJm6 and Diploid AJm7. Nine weeks after vaccination, 14 ISAV‐injected shedder fish that accounted for approximately 10% of the number of fish in the tank, were added. No samples of the fish in this tank were taken during the experiment, but samples were taken at the termination, six weeks after addition of shedder fish.

***Tanks 2 and 3*** were designed similarly as Tank 1 with 30 fish of each group Triploid AJm6, Triploid AJm7, Diploid AJm6 and Diploid AJm7 in each tank. The two tanks were replicates and designed for sampling regarding susceptibility and protection against ISAV between vaccinated groups. Six fish were sampled per group at 9, 11, 13 and 15 wpv.

***Tank 4*** was designed as an infection experiment to estimate differences in susceptibility to ISAV between non‐ISA‐vaccinated diploid and triploid fish. The fish groups, 40 fish per group, were vaccinated with AJm6. Nine weeks after vaccination, ISAV shedders making up 10% of the number of fish in the tank, were added. Samples were taken weekly for five weeks from six fish per group until the termination of the experiment.

#### 2.2.3 Samples

Before vaccination, which took place on Day 0, samples of heart, spleen, kidney and blood from six fish were collected and fixed in RNAlater. Similar samples were taken at the time points described for the individual tanks. Whole blood samples were collected, and the cell pellet and plasma were separated by centrifugation. Weight and length of the fish were measured at all samplings.

#### 2.2.4 RNA isolation and RT‐qPCR

To estimate the level of viral RNA and relative expression of immune response genes, RT‐qPCR was run using samples from six fish from each group from each sampling. Total RNA was extracted from 30–40 mg heart and kidney tissue using the RNeasy Mini kit and QIAcube System (Qiagen) as described earlier (Aksnes et al., 2021). The concentration of RNA was determined by spectrophotometry using the Nanodrop ND1000 (Nanodrop Technologies, Wilmington, USA). For each sample, 750 ng of total RNA from kidney samples and 375 ng from heart were subjected to cDNA synthesis using Quantitech® Reverse Transcription (Qiagen); in a total volume of 20 ul. The TaqMan assay (PE Applied Biosystems) was used for qPCR with an input of 5 μl diluted cDNA (equivalent to 15 ng RNA used in cDNA synthesis) per reaction in a total reaction volume of 13 μl. Primers and probe targeting ISAV genomic segment 8 were used as described earlier (Olsen et al., 2012). The qPCR reaction conditions were 300 nM primer, 200 nM probe, 6.5 μl TaqMan®.

Universal PCR Master Mix (Thermo Fisher Scientific, Waltham, MA) and 0.46 μl RNase‐free water. The cycling parameters were 50°C/2 min and 95°C/10 min, followed by 40 cycles of 95°C/15 s, 58°C/15 s and 60°C/1 min in an AriaMx (Agilent, Santa Clara, CA, USA).

The relative expression of IFNα, Mx and viperin in kidney were examined RT‐qPCR at 10, 11, 12, 13 and 14 wpi. Primer and probe sequences are listed in Table 1.

**TABLE 1:** Primer and probe sequences of the measured innate immune response genes. (FAM is a reporter dye, TAMRA is a quencher for FAM, MBNFQ is minor groove binder non‐fluorescent quencher)

Target	Primer/Probe	Concentration	Sequence (5′‐3′)
Mx	Fwd Rev Probe	300 nM 200 nM	GATGCTGCACCTCAAGTCCTATTA CACCAGGTAGCGGATCACCAT 6‐FAM‐CTGATCAGCCAAACGTTGACTGGATATCCT‐TAMRA
Viperin	Fwd Rev Probe	900 nM 200 nM	CCGGAAGTACAAAGTGGCATTCAAA CTGGTCACTGATGTTTTCTCTCATGT 6‐FAM‐TTAACTCTGTGATCAACACCT‐MGBNFQ
IFNα	Fwd Rev Probe	300 nM 200 nM	ACTGAAACGCTACTTCAAGAAGTTGA GCAGATGACGTTTTGTCTCTTTCCT 6‐FAM‐CTGTGCACTGTAGTTCA‐MGBNFQ

The elongation factor (EF1αb) was used as the reference gene (Lovoll et al., 2011). The cycling parameters were 50°C/2 min and 95°C/10 min, followed by 40 cycles of 95°C/15 s, 58°C/15 s and 60°C/1 min. The Cq for EF1αb in kidney was stable in the range 15.2–15.3, and in heart at 16.2–16.3, and the expression of ISAV RNA is therefore, for simplicity, given as a Cq value.

#### 2.2.5 Statistical analysis

For the experimental data, one‐way ANOVA was performed using JMP Pro 15 for windows (JMP Software, Marlow, United Kingdom). The significant level for rejection of null hypothesis (H_0_) was set at probability value (*p*) < .05. The differences of the RT‐qPCR results were analysed statistically using Wilcoxon matched pairs signed rank test due to the small sample size (*n* = 6). For field data odds ratio for primary ISA outbreak at a site were analysed in Stata using exact logistic regression test for triploid fish versus diploid fish (Stata SE/15; Stata Corp. LLC, TX). Similarly, exact logistic regression was used to analyse odds for SA in triploid versus diploid cages in sites with primary outbreaks.

For the challenge study in tank 1, mortality was used as endpoint. Differences between groups were assessed using a Kaplan–Meier survival analysis followed by a Mantel Cox log‐rank test. To prove vaccine protection, the AJm7 vaccinated groups had to show significant reduced mortality compared to their respective control group, i.e., AJm6 vaccinated groups. If significant protection of vaccination was observed, similar analyses were performed between triploid and diploid fish groups to assess whether these fish populations showed different protection.

## 3 RESULTS

### 3.1 Field studies

In the period 2015–2020 there were 12 confirmed ISA outbreaks within the company's sea sites. In addition, there were two suspected cases where ISAV‐HPRΔ was detected once but ISA was not confirmed in the consecutive testings. ISA occurred in a total of 51 cages in these outbreaks, when including the two suspected cases (one cage each). The proportion of cages with ISA and triploid fish was 25 out of 123 cages (20.3%), while the corresponding proportion for diploid fish and ISA was 26 out of 305 (8.5%). Nine of the 12 confirmed ISA outbreaks were diagnosed more than 12 months after the fish had been put to a sea site. Basic data per year are given in Table S2 (Supplementary information). Three outbreaks were related to smolts that were infected at the smolt plant or immediately after sea transfer.

### 3.2 Primary ISA outbreaks are more prevalent in triploid fish

Based on phylogenic analysis and tracing of infection, the probable origin of the 12 confirmed ISA outbreaks and the two suspected were categorized as primary or secondary at site level. One outbreak in diploid fish was classified as primary, while six outbreaks were assessed as primary in triploid fish (Table 2). The odds ratio for primary ISA outbreak at site level in triploid fish versus diploid were found to be 9.4 (*p* = .047) with confidence interval 1.02–464.

**TABLE 2:** Primary outbreaks of ISA (sites) of the 57 sea transfers of one commercial company of triploid and diploid fish in the period 2015–2019

	Triploid	Diploid	Total
Primary ISA	6	1	7
Not primary ISA	19	31	50
Total	25	32	57

**FIGURE 2:**
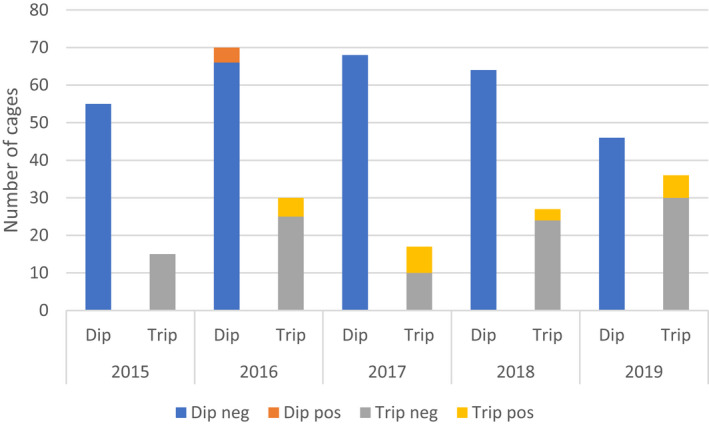
Number of cages per year. Spilt in cages with diploid and triploid fish. Primary ISA outbreaks are shown in yellow/orange.

The distribution of cages per year with or without primary ISA was divided into diploid and triploid (Figure 2). The proportion of cages involved in the six primary ISA outbreaks in triploid fish was 21 of 36 cages (58.3%), while the corresponding proportion for diploid fish was 4 of 14 cages (28.6%). The result were not statistically significant, but indicates an increased odds of 3.4 (*p* = .11) for ISA in triploid cages on sites with primary ISA (confidence intervals 0.8–17.9).

### 3.3 Triploid fish are more susceptible to ISA

There were two cases where the difference between triploid and diploid fish regarding susceptibility to ISA was clearly demonstrated. One example was a primary outbreak in 2020 in a fjord system with three separate production sites. These three sites held a total of 15 cages of which four had triploid fish, and all these four cages were in one of the three production sites. The primary outbreak started in these four cages with triploid fish and developed into clinical disease. In the diploid fish at this site, which was kept in only one separate cage, there was no detection of ISAV‐HPRΔ. The two other sites in this fjord system held only diploid fish and each had just a single episode of ISAV‐HPRΔ detection when several samples were positive, but there was no clinical disease, i.e., these were suspected cases. The fish on these sites were negative on the subsequent sampling, performed to confirm the positive test results, and negative on all monthly samplings that were implemented until slaughter. The sequences of the HE gene of viruses from these three sites of the fjord system were identical.

**FIGURE 3:**
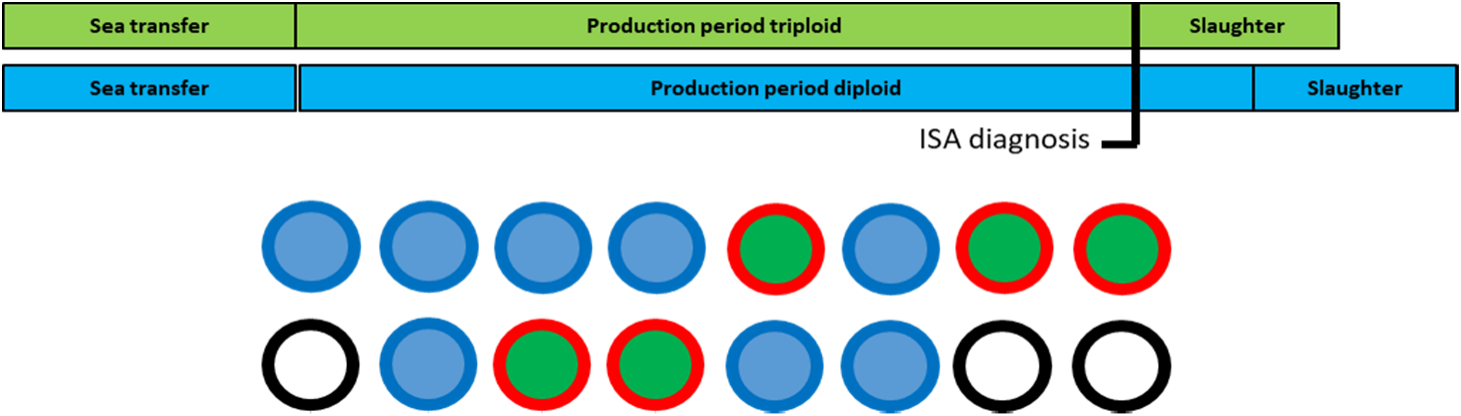
A specific field outbreak of ISA. ISA was confirmed between 9 and 12 months after sea transfer. All five cages with triploid fish became ISA positive, while the eight cages with diploid fish did not. Blue = diploid fish. Green = triploid fish. White = empty cage. Red perimeter = ISA confirmed. Blue perimeter = ISA not confirmed.

In the second example fish were transferred to a sea site of 13 cages where five cages held triploid and eight held diploid fish. ISA was confirmed between 9 and 12 months after sea transfer in all five cages with triploid fish, but not in any of the eight cages containing diploid fish (Figure 3). The triploid smolts originated from three smolt farms, the diploid smolts came from three smolt farms of which one smolt farm was common for both diploid and triploid fish.

### 3.4 Susceptibility and vaccination trials

#### 3.4.1 Equal susceptibility to ISAV for non‐ISA‐vaccinated diploid and triploid fish experimentally

**FIGURE 4:**
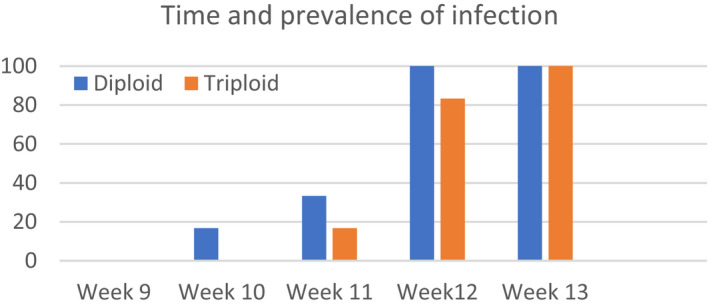
Tank 4. Time of first detection of ISAV in Ajm6 vaccinated groups (*n* = 40 per group, six fish sampled per group per sampling).

Tank 4 was designed to estimate differences in susceptibility to ISAV between non‐ISA vaccinated diploid and triploid fish. There was a tendency that the virus was detected in diploids before triploids, with one more ISAV positive sample out of six samples at 10, 11, and 12 wpv in diploids (Figure 4).

**FIGURE 5:**
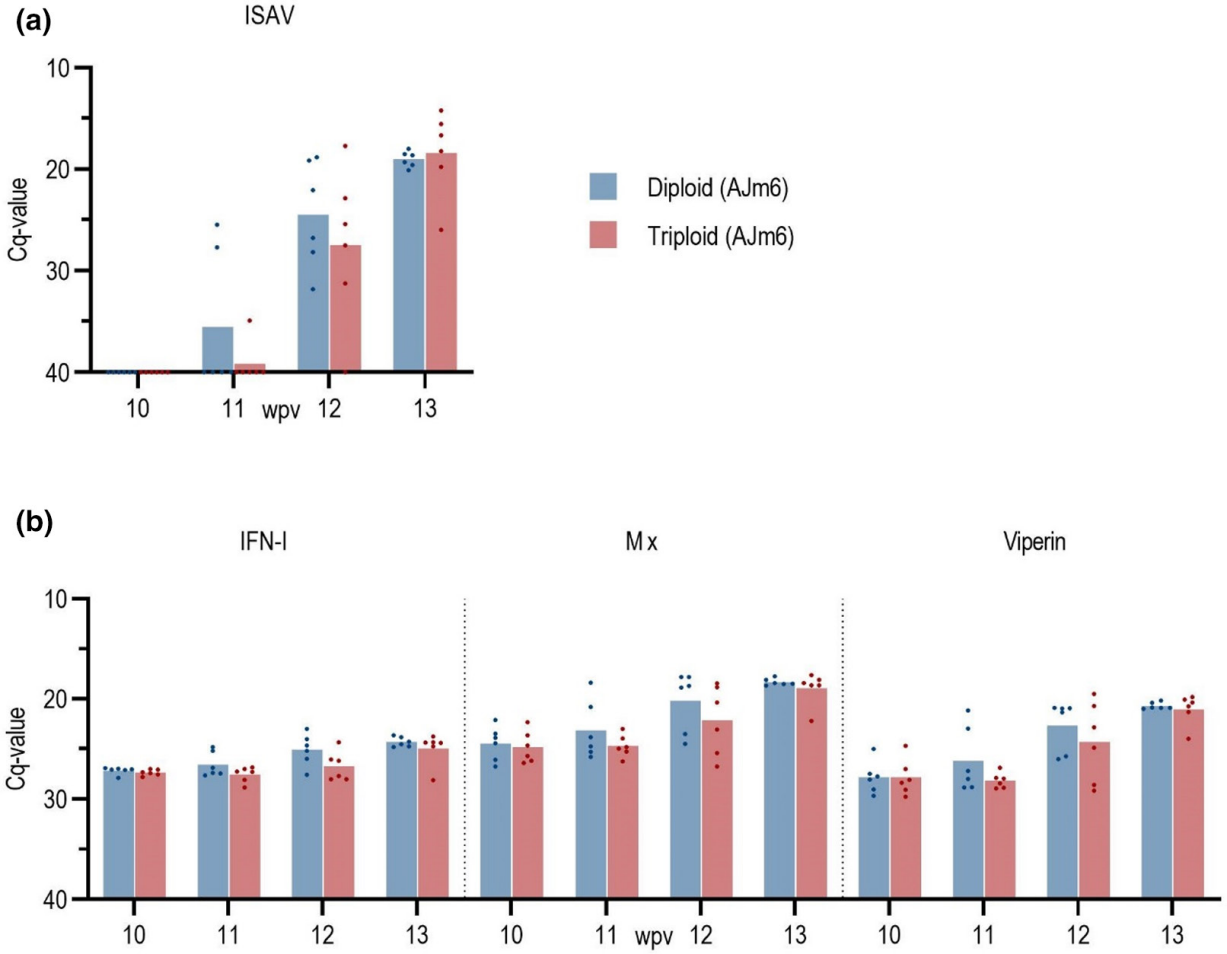
Expression of (a) ISAV and (b) Mx, Viperin, IFN‐1 in kidney of diploid and triploid fish (Tank 4). Dots represent individual samples, bars represent average. Shown as Cq values.

There was no statistically significant difference in viral load, measured as viral RNA, between diploid and triploid groups at any of the sampling times i.e., at week 12 the Cq of groups Diploid AJm6 and Triploid AJm6 were 24.5 ± 5.0 and 26.0 ± 6.6; and at week 13 the Cq were 19.0 ± 0.7 and 18.4 ± 3.8, respectively (Figure 5a). This differs from Tank 2 and 3 where there was significantly (*p* < .05) more virus in Triploid AJm6 than in Diploid AJm6.

#### 3.4.2 Innate immune responses in diploid and triploid fish to ISAV are similar

The innate antiviral response to ISAV in non‐ISA‐vaccinated diploid and triploid fish was assessed by measuring the relative expression in kidney of the genes IFNα, Mx and viperin, normalized to reference gene elongation factor EF1αb. The expression levels of these genes including the elongation factor reference gene before the fish became infected with the ISAV were almost identical for diploid and triploid fish, with Cq value 24.44 and 24.48 for Mx; 27.81 and 27.81 for Vip; 27.14 and 27.24 for IFN‐1; and 15.08 and 15.14 for EF1αb, respectively.

After infection with ISAV the expression of Mx, Vip and IFN‐1 genes increased following the load of viral RNA, with only minor difference in expression levels between diploid and triploid groups (Figure 5b).

#### 3.4.3 Vaccination protects against mortality

The mortality of the virus shedders started ten days after being added to the Tank 1 and they were all dead 15 days after addition. Fish in the vaccinated groups started to die on day 22–23 after the addition of the virus shedders.

**FIGURE 6:**
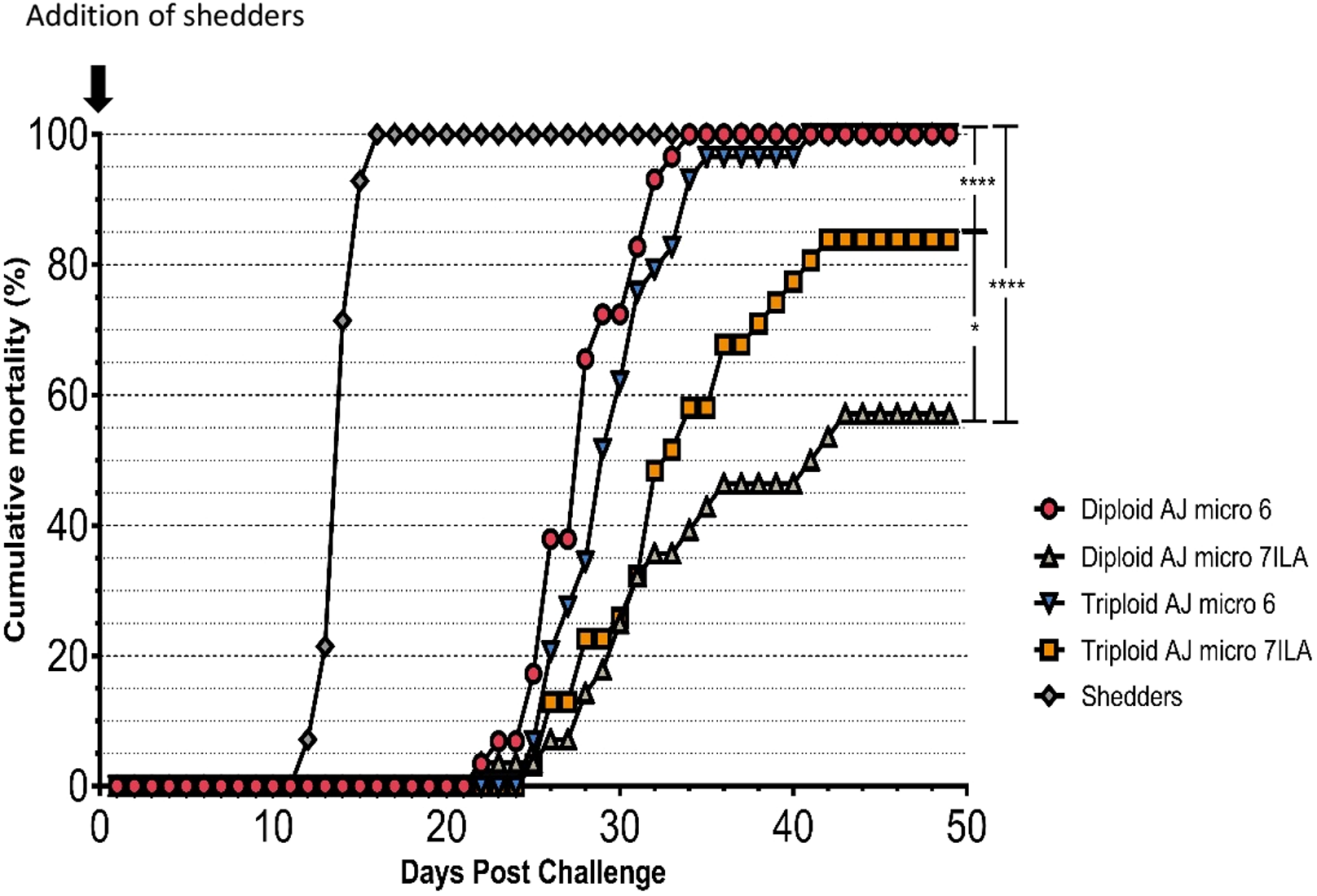
Accumulated mortality in Tank 1 after addition of shedder fish. Statistical analyses were performed by plotting the survival curve for the individual experimental groups (Kaplan–Meier survival analysis) and potentially significant differences between groups were tested by a log‐rank (Mantel‐Cox) test (software: GraphPad Prism v7.03). Only significant analyses are depicted. *N* = 30 fish per group. *p < .05, ****p < .0001.

In the groups Diploid AJm6 and Triploid AJm6, i.e., the groups that received the vaccine without a specific ISA component, the mortality was 100% (Figure 6).

In the groups Diploid AJm7 and Triploid AJm7, i.e., the groups that received the vaccine with a specific ISA component, the cumulative mortality was 57% and 82%, respectively. For both groups, mortality was statistically significantly lower (*p* < .0001) than for the groups Diploid AJm6 and Triploid AJm6 (Figure 6). That is, the vaccine had a statistically significant protective effect against mortality for both diploid and triploid groups.

We found that the difference in mortality between the groups Diploid AJm7 and Triploid AJm7 was also statistically significantly different (*p* < .05) (Figure 6) with a better effect in the diploid group. This may indicate a difference in the effect of vaccination, and/or a more severe ISAV infection in the triploid fish.

#### 3.4.4 Are viral RNA loads higher in non‐ISA‐vaccinated triploid fish?

**FIGURE 7:**
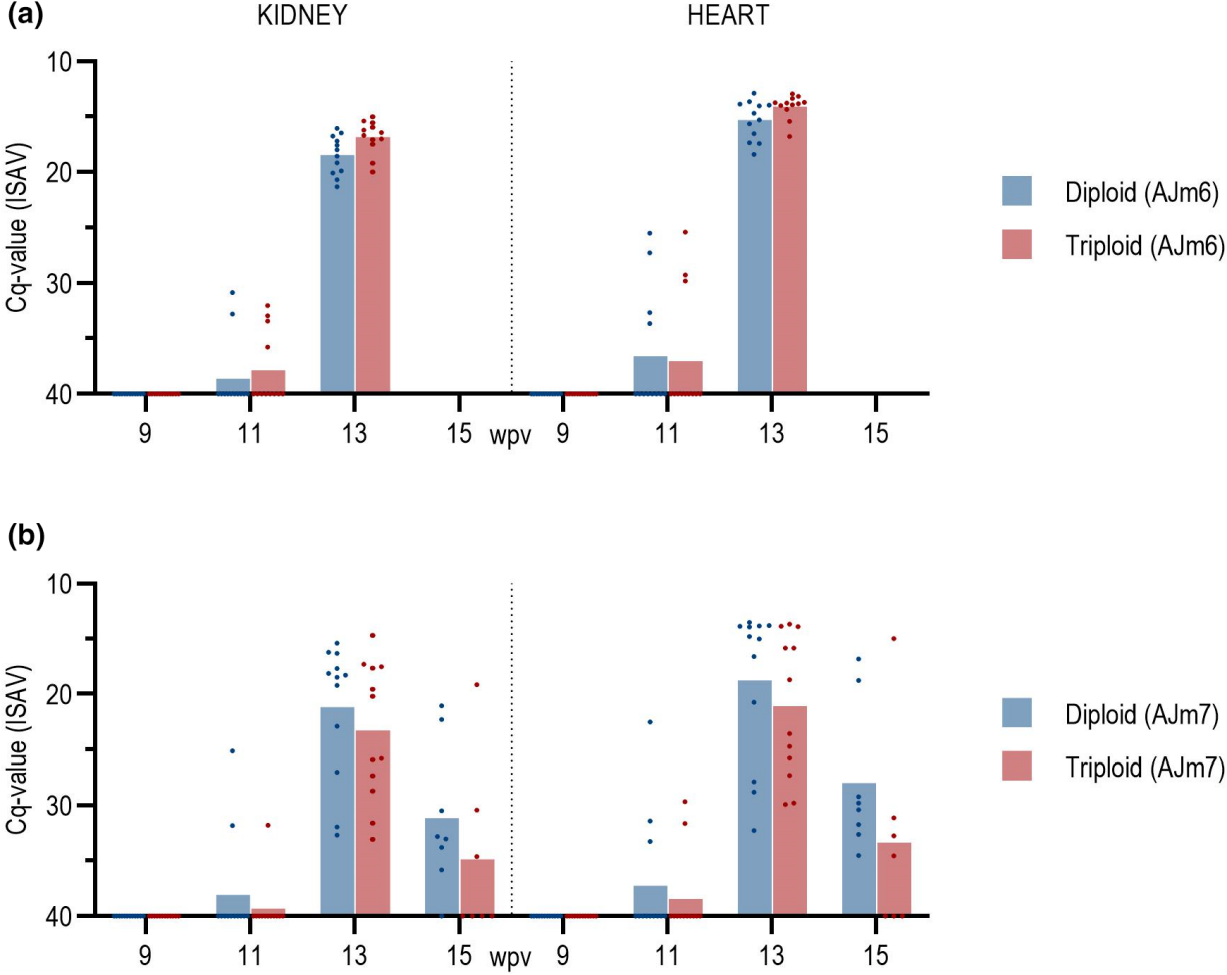
Cq values for ISAV in heart and kidney tissue of diploid and triploid fish, (a) vaccinated without ISAV component (AJm6) or (b) with ISAV component (AJm7). Dots represent individual samples, bars represent average.

At the peak of the infection at 13 weeks post vaccination (wpv), i.e., four weeks after addition of shedder fish, there was significantly more viral RNA (*p* < .05) in Triploid AJm6 than in Diploid AJm6 in both heart and kidney in Tanks 2 and 3 (data not shown). This indicates that more viral RNA is produced in non‐ISA‐vaccinated triploid than in non‐ISA‐vaccinated diploid fish. The amount of viral RNA was very high with Cq ≈ 13–14 in heart at this sampling time, as was reflected by that all the fish in the Ajm6 groups had died by week 15 (Figure 7). As mentioned above, there was no statistically significant difference in viral load, measured as viral RNA, between Triploid AJm6 than in Diploid AJm6 groups in Tank 4. The infection dynamics might have been different in these tanks and could contribute to the different findings, so we could not conclude if viral RNA loads were different in non‐ISA‐ vaccinated diploid versus triploid fish.

#### 3.4.5 Viral RNA loads are lower in ISA‐vaccinated fish

At the peak of the infection, there was significantly more viral RNA in heart and kidney in the non‐ISA‐vaccinated groups Diploid AJm6 and Triploid AJm6 compared to the ISA‐vaccinated Diploid AJm7 and Triploid AJm7 (*p* = .0088). There were no significant differences in the amount of viral RNA between Diploid and Triploid AJm7 groups (Figure 7).

#### 3.4.6 Viral RNA loads are higher in heart than in kidney for all groups

At the peak of the infection at 13 wpv there was statistically significant (*p* < .00001) more virus in the heart than in the kidney for all groups in Tanks 2, 3 and 4 (Figure 7).

## 4 DISCUSSION

The possible negative impact that escaping fish may have on wild fauna is a major concern of Atlantic salmon farming. Triploid fish, being infertile, has been introduced to reduce the genetic impact escapees may have on wild populations of Atlantic salmon. In the last years there has been an increase in the number of ISA outbreaks, mainly in the northern parts of Norway that also is the area where farming of triploid fish has been licensed. The present study investigated the suspected increased susceptibility of triploid Atlantic salmon to ISA.

In the field part of this study, we found an increased risk for primary outbreaks of ISA with a 9.8 odds ratio for primary outbreaks in triploid fish versus diploid fish at site level, and an estimated tendency of 3.4 increased odds for ISA outbreak in triploid fish at cage level in sites with primary ISA. This suggests that triploid fish have an increased risk for both primary outbreak at the site and further development of ISA within the infected site. There were examples of outbreaks at production sites where ISA was diagnosed in the cages with triploid fish and not in the cages with diploid fish. The lack of detection of ISAV‐HPRΔ in diploid fish in neighbour cages to infected triploid fish indicated a lower susceptibility to the infection in diploids. However, some of the ISA cases in diploid populations in this study were due to transmission of ISAV from primary outbreaks in triploid fish. The number of observations in a field study like this will be limited and hence the confidence intervals are large. However, the effect size is large with nearly 10 times increased odds of ISA in triploid fish which strengthens the findings.

Primary outbreaks may be difficult to prevent by biosecurity procedures apart from vaccination because the molecular mechanisms of the transition to ISAV‐HPR0 from ISAV‐HPRΔ are not known and thus not possible to predict. In the experimental ISA vaccine‐ and challenge part of this study, we found that the ISA‐vaccinated groups Diploid AJm7 and Triploid AJm7 both had statistically significantly lower mortalities and lower levels of viral RNA than the corresponding non‐ISA‐vaccinated groups. Although the ISA‐vaccinated groups got infected with ISAV after exposure, the results demonstrated that ISA‐vaccination causes significant protection against ISA‐induced mortality and decreases the load of viral RNA, and thus confirmed that vaccination reduces the potential for spread of infectious virus.

For the ISA vaccinated groups, the survival rate for the Diploid AJm7 was significantly higher than for Triploid AJm7, indicating that vaccination had better effect and/or the infection was better tolerated by diploid than in triploid fish. However, there was no significant difference in the amount of viral RNA between ISA‐vaccinated diploid and triploid fish groups. Even though RT‐qPCR detects both viral mRNA and cRNA in addition to the viral genome and the amount of ISAV RNA does not necessarily mirror the amount of infectious virus, the viral RNA levels did not indicate differences in the viral kinetics between ISA‐vaccinated diploid and triploid fish.

A challenge model comparing vaccinated and non‐vaccinated fish will never fully correspond to the conditions in the field. We tried to adjust the infection pressure by performing a preliminary experiment to estimate a suitable virus dose for injection. However, the amount of infectious virus in the tank water is hard to predict for a cohabitation challenge, especially late in the experiment when the cohabitants themselves start to shed virus. The mortality rate in the AJm7 vaccinated group was relatively high, which may be attributed to the infection pressure in the tanks being too high for vaccination to provide adequate protection.

The fish were smoltified, and stress was experimentally reduced by replacing fresh water with sea water a week before infection. This simulates a natural situation, but since the fish were exposed to virus short after smoltification, it was considered as a relatively tough experimental model for the fish. A cohabitation model where ISAV shedder fish were added to the tanks will mimic the natural course of infection, however, the infection pressure in the tanks, that is the density of infectious virus in the tank water, was assessed as high based on the time course of the infection and possibly higher than what to be expected in the field. The high infection pressure probably contributed to the observed high mortality in the vaccinated groups, thus likely reduced the positive effect of ISA vaccination, and may have masked minor differences between groups.

Triploid fish have three sets of chromosomes and 1.5 times more genetic material than diploid fish. Cells in triploid fish are therefore larger and have a lower surface area to volume ratio, which may impair gas exchange (Riseth et al., 2020). Fish erythrocytes are nucleated, and it can be speculated whether the larger blood cells in triploid fish result in poorer blood flow in peripheral capillaries and contribute to poorer wound healing. Earlier it has been found that triploid Chinook salmon (*Oncorhynchus tshawytscha*) have reduced oxygen‐carrying capacity of the blood (Bernier et al., 2004). Experimental findings have shown that triploid salmon are more sensitive to low oxygen saturation at high water temperatures (Hansen et al., 2015), and that triploid salmon have reduced gill surface (Sadler et al., 2001). In a previous study, it was found that triploid salmon in northern parts of Norway released into the sea in November–December had a higher incidence of skin ulcers (Stien et al., 2019), which is in line with observations of fish health professionals with long experience with triploid fish that observe more winter wounds in triploid than diploid fish (L. Martinsen, personal communications) It has also been noted that triploid fish have a higher prevalence of melanin spots in muscles (Larsen et al., 2014). The results of our study support the assumption that triploid fish are generally more susceptible to disease than diploid fish.

In our data 9 of the 12 confirmed ISA outbreaks occurred more than 12 months after transfer to sea, which indicated that long production time at sea increases the risk to develop ISA. This is in line with earlier findings where long production period in sea was associated with increased risk of primary ISA outbreak (Lyngstad et al., 2018). This was explained with long production period causing increased probability of exposure to both ISAV‐HPR0 and ISAV‐HPRΔ.

At the peak of the infection in the experimental challenge, we found more viral RNA in the heart than the kidney in both diploid and triploid fish. The virus replicates primarily in endothelial cells (Aamelfot et al., 2014), and the findings indicate that samples from heart contain more remnants from endothelial cells than samples from kidney do.

Susceptibility to various infectious diseases has been little studied for triploid fish, but the published studies have presented only minor differences between diploid and triploid fish. In our experiments, we found no differences in the expression level of a limited selected number of innate immune genes between diploid and triploid fish. This is consistent with a previous study done in Chinook salmon (*Oncorhynchus tshawytscha*), where it was concluded that triploid fish had the same level of gene transcription as diploid fish, except when the fish were stressed, where triploid fish showed impaired transcription (Ching et al., 2010).

In the field study there were sites where ISA was detected in cages with triploid fish, but not in cages with diploid fish. There were also examples of sites where cages with triploid fish were infected while cages with diploid fish in nearby farms in the same fjord system only had a single episode of ISAV‐HPRΔ detection and then became negative on subsequent samplings, and where the HE gene sequences were identical in the triploid and diploid fish. Both these examples, together with the finding of an increased risk for primary ISA outbreaks in triploids found in general, strongly indicate that triploid fish are more susceptible to ISAV. However, in the experimental study we found only minor differences in the susceptibility to infection between non‐ISA‐vaccinated diploid and triploid salmon, as assessed by the time of first detection of infection after exposure to virus shedders and by the number of infected fish at the different samplings. There was even a slight trend that diploid fish got infected earlier. Although the infection pressure in the experimental challenge may have contributed to mask minor differences between groups the discrepancy between the findings of the field and the experimental studies regarding susceptibility was unexpected. Could the strains of ISAV be of importance for these results? In the field, the virus in the two examples mentioned above emerged in triploid fish and was found to be more susceptible to triploids than diploids, while the ISAV strain AL V321 that was used in the experiments was originally isolated from diploid fish and had regularly been used in experiments in diploids and thus could be called a strain adapted to diploid fish. It could therefore be speculated if the affinity of these ISAV strains differed between diploid and triploid fish and that this may have contributed to the inconsistent findings.

Furthermore, the experimental and field observations did not necessarily describe the same infection mechanisms. The experimental infection trial described susceptibility to and consequences of an infection with a known virulent variant of ISAV. Primary outbreaks with ISA in the field, on the other hand, are not infection with a known virulent variant of the virus, but rather the emergence of a virulent ISAV variant or infection with such a variant transmitted from an unknown source. In short, the experimental infection therefore trial did not mirror the emergence of a primary ISA outbreak. ISAV replicates in the cell nucleus and this replication depends upon the cell's transcription machinery. Our experimental findings show that ISAV utilizes the transcription machinery just as well in triploid as in diploid cells. The molecular mechanisms behind the emergence of a virulent variant of the ISA virus are unknown but take place in the cell nucleus. It could be speculated that the differences in the microenvironments in triploid versus diploid cell nuclei can have an impact on the potential for the development of virulent ISAV variants, and that this more easily occur in triploid cells.

## 5 CONCLUSIONS

We found that triploid Atlantic salmon may have increased risk of primary field outbreaks of ISA. But we found no indications for a difference in susceptibility to an ISAV virulent strain for non‐ISA vaccinated diploid and triploid fish in an experimental setting.

There was better survival, and lower viral RNA load, in ISA‐vaccinated than in non‐ISA‐vaccinated fish in both diploid and triploid fish. Vaccinated fish were infected with the virus after exposure.

The increased risk of ISA in triploid fish should be part of an evaluation of the costs and benefits of using triploid salmon in farming. It could be recommended triploid and diploid fish are kept in biosecure separated sites or are not farmed at all.

## AUTHOR CONTRIBUTIONS

Conceptualization: AA, ER; Methodology: AA, MMS‐K, SB; formal analysis: AA, MMS‐K, SB; investigation: AA, LM, TB, MMS‐K, AS, SG, ER; data curation: AA, MMS‐K, SB, ER; writing—original draft preparation: ER, AA; writing—review and editing: AA, LM, TB, MMS‐K, AS, SG, SB, ER; project administration: AA, LM, MMS‐K, ER; funding acquisition: AA, MMS‐K, ER. All authors have read and agreed to the published version of the manuscript.

## CONFLICT OF INTEREST

LM is an employee of NRS, and MMS‐K and ASL are employees of Pharmaq. They declare no conflict of interest. The other authors declare no conflict of interest. The funders had no roles in the design of the study, in the collection, analyses, or interpretation of data, in the writing of the manuscript, or in the decision to publish the results.

## Supporting information


Table S1
Table S2

## Data Availability

The research data acquired in this work are open and available. Norwegian University of Life Sciences adheres to the FAIR‐principles, **F**indable, **A**ccessible, **I**nteroperable and **R**eusable.
