# Supplementary material for: Triploid Atlantic salmon (Salmo salar) may have increased risk of primary field outbreaks of infectious salmon anaemia
Source: J Fish Dis. 2022 Aug 1;45(11):1733–43. doi: 10.1111/jfd.13695 (PMC9805046; doi:10.1111/jfd.13695)
Supplement: Supplementary file 1 — Table S1 Table S2 [file JFD-45-1733-s001.docx]

**Appendices**

**Table S1.** ISA outbreaks within the company in the period 2015-2020.

| ***Locality*** | ***Number of months***  ***after sea transfer*** | ***Year of detection*** | ***Outbreak in diploid or triploid*** |
| --- | --- | --- | --- |
| A | 3 | 2015 | Diplo |
| B | 2 | 2015 | Diplo |
| C | 13 | 2017 | Diplo |
| D | 13 | 2017 | Diplo |
| E | 15 | 2017 | Triplo |
| F | 13 | 2018 | Triplo/diplo |
| G | 6 | 2019 | Triplo |
| H | 18 | 2019 | Triplo |
| I | 20 | 2020 | Triplo |
| J | 20 | 2020 | Diplo |
| K | 14 | 2020 | Triplo/diplo |
| L (suspected) |  | 2020 | Diplo |
| M (suspected) |  | 2020 | Diplo |
| N | 12 | 2020 | Triplo |

**Table S2**. Primary ISA outbreaks per cage per year, split in diploid and triploid

| **ISA** | **Dip-loid** | **Trip-loid** | **Sum** | **ISA, In total (%)** | **ISA,Diploids (%)** | **ISA,Triploids (%)** | **Diploid**  **cages (%)** | **Triploid**  **cages (%)** |
| --- | --- | --- | --- | --- | --- | --- | --- | --- |
| 2015 | 55 | 15 | 70 |  |  |  | 78.6 | 21.4 |
| Primary ISA | 0 | 0 | 0 | 0 | 0.0 | 0.0 |  |  |
| 2016 | 70 | 30 | 100 |  |  |  | 70.0 | 30.0 |
| Primary ISA | 4 | 5 | 9 | 9.0 | 5.7 | 16.7 |  |  |
| 2017 | 68 | 17 | 85 |  |  |  | 80.0 | 20.0 |
| Primary ISA | 0 | 7 | 7 | 8.2 | 0.0 | 41.2 |  |  |
| 2018 | 64 | 27 | 91 |  |  |  | 70.3 | 29.7 |
| Primary ISA | 0 | 3 | 3 | 3.3 | 0.0 | 11.1 |  |  |
| 2019 | 46 | 36 | 82 |  |  |  | 56.1 | 43.9 |
| Primary ISA | 0 | 6 | 6 | 2.4 | 0.0 | 16.7 |  |  |
| **2015-19** | **305** | **123** | **428** |  |  |  | **71.3** | **28.7** |
| **Primary ISA** | **4** | **21** | **25** | **4.9** | **1.3** | **17.1** |  |  |
